# Women’s Preferences and Willingness to Pay for AI Chatbots in Women’s Health: Discrete Choice Experiment Study

**DOI:** 10.2196/67303

**Published:** 2025-06-10

**Authors:** Jing Wang, Hewei Min, Tao Li, Jiaheng Li, Yang Jiang, Jingbo Zhang, Yibo Wu, Xinying Sun

**Affiliations:** 1 School of Public Health, Peking University, 38 Xueyuan Road, Haidian District, Beijing, 100191, China, 86 13691212050; 2 Department of Gynaecology and Obstetrics, The Fourth Central Hospital of Baoding City, Hebei, China; 3 School of Basic Medical Sciences, Hebei University, Hebei, China; 4 Jitang College, North China University of Science and Technology, Hebei, China; 5 Center for Evidence-Based Chinese Medicine, Beijing University of Chinese Medicine, Beijing, China

**Keywords:** women’s health, artificial intelligence chatbots, discrete choice experiment, willingness to pay, health education

## Abstract

**Background:**

Over 96% of adult women face health issues, with 70% experiencing conditions like infections. Mobile health education is increasingly popular but faces challenges in personalization and readability. Artificial intelligence (AI) chatbots provide tailored support, and a discrete choice experiment can help in understanding user preferences to improve chatbot design.

**Objective:**

This study aims at exploring the preferences of women toward AI chatbots to improve health education communication and user experience.

**Methods:**

A discrete choice experiment was conducted, identifying 6 main attributes of AI chatbots: response accuracy, legibility, service cost, background information collection, information utility, and content provision. A total of 957 female participants from a hospital in Hebei Province participated, choosing between 2 hypothetical chatbots or opting for neither (a no-choice option). The conditional logit model was used to estimate user preferences.

**Results:**

A total of 957 participants were included in the analysis. The results showed that participants preferred a chatbot with 100% response accuracy (β=0.940, *P*<.001; 95% CI 0.624 to 1.255), very easy to understand information (β=0.907, *P*<.001; 95% CI 0.634 to 1.180), a service fee of CN ¥0/month (β=−0.095, *P*<.001; 95% CI −0.108 to −0.082; a currency exchange rate of US $1=CN ¥7.09 was applicable), practical information utility (β=1.085, *P*<.001; 95% CI 0.832 to 1.338), and provision of disease-related knowledge (β=0.752, *P*<.001; 95% CI 0.485 to 1.018). Whether or not to allow the collection of background information (only question and answer information) has no significant impact on women’s choice preferences. Additionally, participants were willing to pay an additional CN ¥9.916 (95% CI 6.843 to 12.292) for 100% response accuracy, CN ¥9.567 (95% CI 6.843 to 12.292) for “very easy to understand” information, and CN ¥11.451 (95% CI 8.704 to 14.198) for the “very practical” information utility. Additionally, they were willing to pay CN ¥7.931 (95% CI 4.975 to 10.886) for “knowledge of diseases” compared to “gender knowledge” (CN ¥2.602, 95% CI −0.551 to 5.756). The relative importance of the chatbot attributes indicated that information utility (1.085/3.858, 28.12%) and response accuracy (0.940/3.858, 24.37%) were the most influential factors in participants’ preferences.

**Conclusions:**

AI chatbots designed for female users should focus on high response accuracy, clear content, free access, privacy protection, practical information, and disease knowledge to attract users and enhance health education.

## Introduction

According to the United Nations [1], women make up 49.75% of the global population, yet their health needs are often overlooked. In response, the World Health Organization [2] identified 6 key priorities for women’s health in 2021, aiming to address the numerous challenges faced by women and girls in accessing adequate health care. These priorities included ensuring universal access to sexual and reproductive health services and tackling noncommunicable diseases, which remain a leading cause of preventable death among women worldwide (World Health Organization) [2]. The agenda also focused on eliminating violence against women and preventing noncommunicable diseases, including obesity. Furthermore, neglecting women’s health could jeopardize the well-being of future generations.

Compared to other health conditions, issues related to women’s health are generally more preventable and sensitive. Effective health education programs play a key role in preventing these conditions, while accurate health knowledge acquisition helps mitigate disease progression [3]. Combining systematic education with evidence-based knowledge dissemination not only benefits the women themselves but also has significant implications for society as a whole.

There are various methods to implement health education for women. These include regular physical examinations with structured educational components, distributing scientifically validated informational materials, and offering medical consultations integrated with knowledge-sharing sessions. Knowledge dissemination is also achieved through special television programs and radio segments [4] that translate complex medical information into accessible content. With the advancement of technology, the paradigm of health knowledge acquisition has been transforming. Mobile health education, based on apps and social media platforms, facilitates knowledge transfer while developing self-care skills among women [5-10]. In contrast to traditional methods, mobile health education offers greater accessibility, personalization, and cost-effectiveness, overcoming time and geographical barriers [11-13].

Mobile health education still faces challenges in pedagogical design and knowledge delivery. Educational effectiveness depends on content diversity, while knowledge retention requires information accuracy. For educational interventions targeting vulnerable groups, designing pedagogically sound content with actionable knowledge points is crucial. In this context, artificial intelligence (AI) chatbots emerge as a promising solution that may combine digital education with personalized knowledge delivery [14]. AI, which comprises programs and algorithms that perform tasks traditionally requiring human intelligence, is increasingly applied in health care, with potential in both specialized and general settings [15,16]. AI chatbots, as one of the key AI apps, offer digital platforms for delivering health information and services [17]. Using natural language processing [18], these systems convert user inquiries into a machine-readable format, leveraging a broad spectrum of medical data to establish a knowledge base that supports health-related interactions [19]. AI chatbots could offer appropriate health advice and facilitate health discussions, thereby supporting communication and patient empowerment [20]. With the expansion of mobile internet access, AI chatbots are being explored as a practical and accessible tool for women with limited health care access, enhancing patient-centered care and self-management [21]. Their use notably increased during the COVID-19 pandemic, demonstrating some positive support in various health domains [17].

Specifically, AI chatbots have the potential to restructure health education frameworks by providing tailored guidance, while establishing reliable knowledge repositories on public platforms [22,23]. For instance, Rosie, a health education chatbot, is a great example. Rosie aims to provide reliable health information to new mothers, helping them address various issues during pregnancy and postpartum. Through interactions with the community, Rosie has not only enhanced users’ access to health information but also helped to narrow the health disparities between different racial groups to some extent [24]. Maeda et al [25] conducted a randomized controlled trial with 927 women to assess the effects of a fertility education chatbot, finding it significantly improved fertility knowledge and preconception behaviors. The chatbot also reduced anxiety, demonstrating its potential to educate and empower women without increasing stress. Additionally, a review by Kim [26] examined the broader effects of AI chatbots on women’s health, including mental health, cancer self-care, and preconception intentions. The meta-analysis revealed that chatbot interventions significantly reduced anxiety, showing the positive impact of such technologies on physical, physiological, and cognitive health outcomes. This suggests that AI chatbots can serve as an effective tool in addressing various health concerns and providing digital therapeutic support to women.

However, despite these advancements, there has been insufficient exploration of how these AI tools can be customized for specific gynecological needs. AI chatbots that fully consider the needs and preferences of users may better enhance the effectiveness of interventions [27]. Previous studies have shown that people tend to care about the accuracy of medical chatbot responses and are concerned about whether their privacy is being violated [28-30]. A review of mental health chatbots indicated that patients generally find the usefulness and ease of use of these chatbots to be positive, but were less satisfied with the language expression and response quality of the chatbots. Some chatbots were considered slow in response, with answers that were often superficial, brief, and sometimes even confusing [31]. Moreover, another review revealed that the personalization of chatbots, such as in response results, content, user interfaces, delivery channels, and features, was an important factor in improving user satisfaction [32]. In summary, research on AI needs and preferences has mostly focused on the general population or adolescents, with some involvement in the development and research of mental health chatbots [27,31,33,34]. However, most studies have concentrated on English-language chatbots and lack in-depth investigation into preference differences among various demographic groups, which could potentially limit the benefits that different populations can derive from these technologies.

This study aims to explore Chinese women’s preferences regarding the attributes of AI chatbots. To improve user experience and the accuracy of AI chatbot information, it is crucial to understand user preferences. A suitable method for measuring these preferences is the discrete choice experiment (DCE), a robust survey technique that presents respondents with multiple choices among hypothetical treatments [35]. The choices made by respondents are influenced by the alternatives presented. By offering options that consist of various attributes at differing levels and repeatedly asking participants to select their preferred option or the one that maximizes utility [36], we seek to understand how users may value different aspects of AI chatbot use in women’s health. Quantifying these preferences enables the identification of preferred attributes and provides insight into how women perceive and value AI chatbots for their health needs [37].

## Methods

### Study Design

In this study, we investigated women’s preferences for attributes of the AI chatbots using the DCE method. DCE is a questionnaire-based stated preference approach to study people’s preferences for certain characteristics or conditions by asking subjects to make choices about hypothetical scenarios or alternatives they prefer [38,39], DCE can be used to obtain quantitative data about people’s preferences, gain in-depth insights and predictive power, and is often applied in market research, social sciences, medicine, and other fields. Selected attributes in the DCE scheme can contain price attributes for different price levels to estimate willingness to pay (WTP).

### Development of the DCE

In this study, to ensure that the selected attributes comprehensively reflect user preferences for AI chatbots and have broad representativeness, we followed a systematic process involving a preliminary literature review, expert consultations, and a presurvey.

First, we conducted a preliminary literature review by searching multiple academic databases (such as Google Scholar, Web of Science, PubMed, etc) using relevant keywords like “AI chatbots,” “user preferences,” and “digital health,” and focusing on studies published between 2015 and 2022. We selected studies that focused on female participants, including both quantitative and qualitative research that explored the characteristics of AI chatbots and users’ responses to them. This ensured that the literature reviewed was relevant to the female population, providing a solid theoretical foundation for identifying potential attributes that could influence user choices [28,30,40-44]. Based on this review, we moved to the expert consultation phase.

We conducted 3 rounds of expert consultations, with each round having specific objectives and outcomes. In the first round, we brought together a multidisciplinary team of 10 experts from fields such as law, ethics, AI, and health data management. During this round, we presented the initial set of attributes identified from the literature review and asked the experts to review and comment on their relevance, clarity, and comprehensiveness. Their feedback led to the removal of certain attributes that were deemed too general or not actionable. In the second round, the attributes were refined based on the feedback from the first round, and the experts were asked to further discuss the attributes’ levels, ensuring that they were clear and easy to understand for the target audience. This round also involved developing concrete examples for some attributes to help experts understand their real-world applications. In the third and final round, the experts were asked to validate the revised attributes and provide final input on their importance and whether they sufficiently reflected user preferences. The outcome of this round was the identification of 6 core attributes, with final adjustments made to the descriptions and levels to make them as actionable and understandable as possible.

After the expert consultations, we proceeded with a presurvey to test the applicability of the selected attributes within the target group. The survey was conducted with 100 women, who were recruited from a hospital in Hebei Province. The purpose of this presurvey was to gauge how well the attributes were understood, ranked, and valued by potential users. Through this survey, we collected feedback on the clarity of the attribute descriptions and the relative importance of each attribute. The results indicated that some attribute descriptions were unclear or too complex, requiring further revision. Based on the presurvey feedback, we modified the wording of the attributes to ensure that they were easily understandable and accurately captured the participants’ preferences.

Through these three comprehensive validation steps—literature review, expert consultation, and presurvey—we ultimately identified six key attributes that would guide the design of the AI chatbot: (1) response accuracy, (2) legibility, (3) service cost, (4) whether to allow background collection of information (question and answer information only), (5) information utility, and (6) provision of information content.

Each attribute and its level are shown in Table 1.

**Table 1.** Attributes and levels of the chatbot.

Attribute	Attribute level^a^
Response accuracy	60%, 70%, 80%, 90%, or 100%
Legibility	Very easy to understand, easier to understand, harder to understand, or difficult to understand
Service cost (CN ¥)^b^	0/month, 5/month, 10/month, 15/month, or 20/month
Whether to allow the collection of background information (only question and answer information)	Yes or no
Information utility	Very practical, more practical, less practical, or very impractical
Provision of information content	Knowledge of diseases, daily health case, knowledge of maternity, or gender knowledge

a Refers to the different levels or variations of each attribute used in the discrete choice experiment.

b A currency exchange rate of US $1=CN ¥7.09 was applicable.

**Figure 1.**
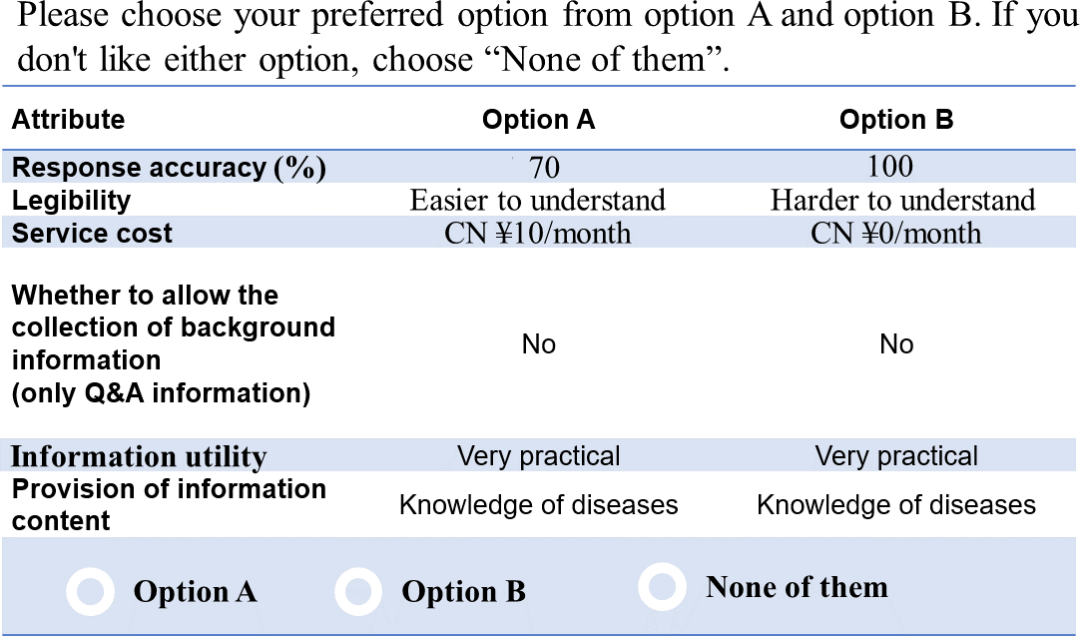
An example scenario of the choice-based conjoint in the questionnaire. A currency exchange rate of US $1=CN ¥7.09 was applicable. Q&A: question and answer.

Based on the identified attribute levels, we created choice sets containing various attribute levels for respondents to select from through orthogonal tests. Given that there are 2 to 5 attribute levels for each of the 6 attributes in our study, using a full factorial design would result in 3200 combinations (5×4×5×2×4×4=3200), which is clearly impractical for generating such a large number of choice sets. Consequently, we used a fractional factorial design to determine the optimal number of choice sets. Based on the principles of orthogonality, balance, and minimal overlap, 25 proposals were ultimately generated. Selecting one of them as a fixed reference, along with the remaining 24 options, forms a total of 24 selection sets. Each selection set contains 3 conceptual options (“choose option A,” “choose option B,” or “choose neither”). Randomly dividing all selection sets into 4 groups, each group consists of 7 selection sets (6 random selection sets and 1 repeated selection set). Respondents selected their preferred combination of attributes for the AI chatbots in each scenario or opted for none, thereby minimizing bias resulting from forced choices. According to the sample size formula for DCEs: n=(500c)/(ta), where “c” represents the largest number of levels for an attribute, “t” denotes the number of choice sets in a block, and “a” indicates the number of alternatives. For this study, the values were as follows: “c” was set to 5, “t” was set to 6, and “a” was set to 2. Therefore, the minimum required sample size for this study was determined to be 209 participants. Figure 1 shows an example of a DCE program.

### Study Population and Data Collection

This study began in December 2022 and involved a questionnaire survey of women at a hospital in Hebei Province, lasting for 2.5 months. The recruitment period started on December 20, 2022, and ended on February 15, 2023. Convenience sampling was used, with gynecologists recruiting eligible female patients from both the gynecology outpatient department and the gynecology inpatient ward. Research staff explained the purpose of this study to eligible women and invited them to participate. Research information was collected through electronic questionnaires, which were divided into 2 sections: the first section gathered basic demographic information, including age, education level, and usual residence, while the second section assessed preferences regarding AI chatbots. The electronic questionnaire was distributed via the WJX platform[45]. After patients agreed to participate and completed the informed consent form, they filled out the questionnaire online by scanning a QR code. The inclusion and exclusion criteria are as follows:

The inclusion criteria are as follows: (1) age ≥18 years; (2) sex: female; (3) possesses the nationality of the People’s Republic of China; (4) own a smartphone and have the ability to use WeChat, including following WeChat public accounts; (5) ability to complete web-based questionnaires independently or with assistance from another; (6) possess basic literacy skills to enable normal communication and interaction; and (7) voluntary participation in the study, with willingness to complete an informed consent form.

The exclusion criteria are as follows: (1) individuals with severe cognitive impairments or mental health conditions, (2) individuals who are participating in other similar research projects, and (3) individuals who are unwilling to cooperate.

**Figure 2.**
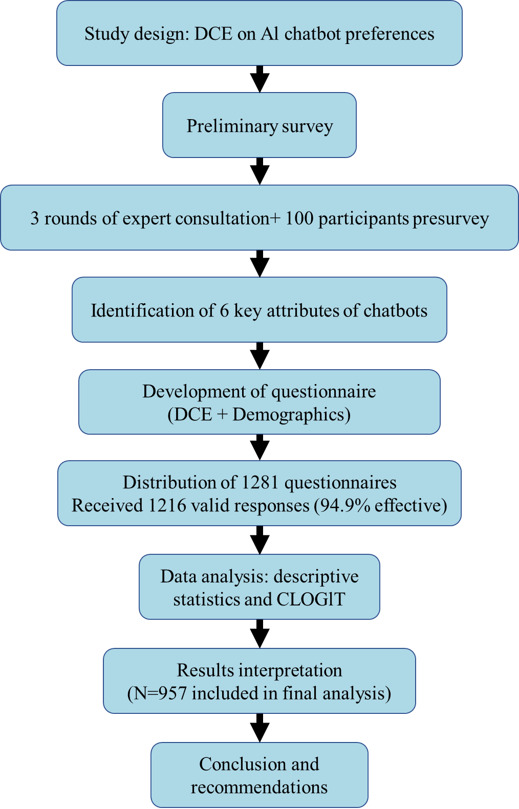
Flowchart of study process. AI: artificial intelligence; DCE: discrete choice experiment; CLOGIT: conditional logit model.

A total of 1281 women were invited to participate, and 1216 women agreed to participate, yielding a participation rate of 94.9%. The final sample size of 957 participants was determined after excluding unqualified questionnaires based on the exclusion criteria. This process is depicted in Figure 2, which illustrates the flowchart of this study’s recruitment and analysis.

### Ethical Considerations

This study has been approved by the Ethics Committee of the Baoding No. 4 Central Hospital (2022013). All study participants have voluntarily participated and signed informed consent forms, ensuring that they are fully aware of this study’s objectives, methods, and any potential risks. This consent was obtained before participation, per the ethical standards for human subject research. As this study involves human participants, appropriate ethical oversight was conducted, and all research activities were approved by the Ethics Committee. To ensure privacy and confidentiality, all study data are anonymized and deidentified to protect the identity of the participants. The data will be stored securely and will only be accessible to authorized personnel involved in this research. No compensation is provided to the participants for their involvement in this study. To maintain the confidentiality and anonymity of the participants, no identifiable images or data of individual participants will be included in this paper or supplementary materials.

### Statistical Analysis

Statistical analyses were performed using IBM SPSS Statistics (version 26.0; IBM Corporation) and Stata (version 15.0; StataCorp LLC). Descriptive statistics of demographic variables were performed by frequency counts (composition ratios). Conditional logit models (CLOGITs) were used to quantify the relative levels of attribute preferences used by the AI chatbots via Stata (version 15.0). Different levels of each attribute were dummy-coded, and one of the levels was set as the reference level. In a DCE study, the calculated results provide important statistical information. These results include coefficients, *P* values, SEs, and 95% CI. We also calculated this study’s participants’ WTP for different attribute levels of the AI chatbots to more intuitively reflect the strength of the respondents’ attribute preferences for the AI chatbots.

## Results

### Characteristics of Respondents

The sociodemographic characteristics of this study’s sample are summarized in Table 2.

The age distribution showed that the largest group was aged between 26 and 35 years (n=431, 45.04%), followed by those aged 36 to 45 years (n=280, 29.26%). For further details on participant demographics, please refer to Table 2.

**Table 2.** General characteristics of the subjects (N=957).

Items	Cases, n (%)
Occupation	
Employed	726 (75.86)
Student	27 (2.82)
Retired	11 (1.15)
Unstable occupation, freelancer, unemployed, and not in employment	193 (20.17)
Location	
Urban	633 (66.14)
Rural	324 (33.86)
Age (years)	
18‐25	159 (16.61)
26‐35	431 (45.04)
36‐45	280 (29.26)
>45	87 (9.09)
Education level	
Junior high school or lower	266 (27.8)
Special school or senior high school	211 (22.05)
Junior college or higher	480 (50.16)
Marital status	
Single	115 (12.02)
Married (including first marriage, remarried, or remarried after divorce)	823 (86)
Divorced	16 (1.67)
Widowed	3 (0.31)
Income^a^^b^	
≤1000	66 (6.9)
1001‐3000	338 (35.32)
3001‐5000	386 (40.33)
>5000	167 (17.45)

a Per capita monthly household income.

b A currency exchange rate of US $1=CN ¥7.09 was applicable.

### Percent Importance of AI Chatbot Attributes

The percent importance reflects the extent to which each attribute influences the differences in preferences. It is calculated based on the range of preference weights across each attribute’s levels, which provides a measure of its impact on decision-making. The sum of all percent importance is 100%, and a higher value corresponds to a greater influence on preferences. The information utility of the chatbot is considered the most important attribute (1.805/3.858, 28.123%), while cost and background information collection are the lowest, at 2.462% (0.095/3.858) and 2.074% (0.080/3.858), respectively.

### DCE Results

The results of the CLOGIT model analysis indicate that the participants exhibited a preference for attributes such as 100% response accuracy, very easy to understand readability, CN ¥0/month (a currency exchange rate of US $1=CN ¥7.09 was applicable) service cost, very practical information utility, and provision of information content as knowledge of the disease in the AI chatbots. Table 3 presents detailed information on the preference attributes and their coefficients.

**Table 3.** CLOGIT^a^ results of participants’ preferences for attributes of AI^b^ chatbots.

Attributes and levels	Coefficient	*P* value	SE	95% CI
Response accuracy (%)				
60^c^				
70	0.561	.001	0.168	0.232 to 0.891
80	0.511	.002	0.167	0.184 to 0.838
90	0.637	<.001	0.161	0.321 to 0.953
100	0.94	<.001	0.161	0.624 to 1.255
Legibility				
Harder to understand^c^				
Difficult to understand.	0.207	.21	0.163	–0.113 to 0.528
Easier to understand	0.725	<.001	0.137	0.456 to 0.993
Very easy to understand.	0.907	<.001	0.139	0.634 to 1.180
Service cost	–0.095	<.001	0.007	–0.108 to –0.082
Whether or not to allow the collection of background information (only question and answer information)				
Yes^c^				
No	0.080	.37	0.090	–0.095 to 0.254
Information utility				
Very impractical^c^				
Less practical	0.180	.24	0.153	–0.121 to 0.481
More practical	0.584	<.001	0.164	0.262 to 0.907
Very practical	1.085	<.001	0.129	0.832 to 1.338
Provision of information content				
Gender knowledge^c^				
Knowledge of maternity	0.247	.11	0.153	–0.141 to 0.447
Daily health care	0.664	<.001	0.137	0.396 to 0.932
Knowledge of diseases	0.751	<.001	0.136	0.485 to 1.018

a CLOGIT: conditional logit models.

b AI: artificial intelligence.

c Reference level.

### WTP Results

WTP is a valid indicator of how much money a person is willing to sacrifice to choose 1 diagnostic attribute level over another. Table 4 presents the participants’ WTP for different attributes of the AI chatbot. The findings indicate that participants are willing to pay more for a women’s health AI chatbot that provides accurate answers, clear content, does not collect background information, has high information availability, and focuses on disease-related knowledge.

**Table 4.** Participants’ WTP^a^ for AI^e^ chatbots.

Attributes and levels	WTP (CN ¥)^c^	95% CI
Response accuracy (%)		
60^d^		
70	5.924	2.628 to 9.22
80	5.389	2.034 to 8.744
90	6.721	3.637 to 9.806
100	9.916	6.843 to 12.292
Legibility		
Harder to understand^d^		
Difficult to understand.	2.188	–1.154 to 5.529
Easier to understand	7.646	4.883 to 10.409
Very easy to understand.	9.567	6.843 to 12.292
Whether or not to allow the collection of background information (only question and answer information)		
Yes^d^		
No	0.839	–1.017 to 2.695
Information utility		
Very impractical^d^		
Less practical	1.9	–1.278 to 5.078
More practical	6.167	2.701 to 9.633
Very practical	11.451	8.704 to 14.198
Provision of information content		
Gender knowledge^d^		
Knowledge of maternity	2.602	–0.551 to 5.756
Daily health care	7.006	4.049 to 9.963
Knowledge of diseases	7.931	4.975 to 10.886

a WTP: willingness to pay.

b AI: artificial intelligence.

c A currency exchange rate of US $1=CN ¥7.09 was applicable.

d Reference level.

## Discussion

### Principal Findings

This study used a DCE to explore women’s preferences for AI chatbots. The results revealed significant variations in user preferences across different chatbot attributes, particularly for information usefulness, response accuracy, readability, and content provision, whereas cost and data collection were of relatively lower importance.

The scalability, accessibility, ease of use, and rapid information dissemination of AI chatbots offer supplementary benefits to public health efforts [46], addressing issues such as capacity constraints, social distancing requirements, and misinformation [47]. Research suggests that people sometimes prefer interacting with AI chatbots over doctors in certain contexts, as these chatbots can respond more quickly, provide high-quality feedback [48], and offer empathetic interactions. The application of AI in medicine is expanding across various domains, including medical image analysis [49], drug interaction detection [50], high-risk patient identification [51], and medical record coding [52]. In health care education, tools like ChatGPT have been used to facilitate personalized learning, encourage critical thinking, and support problem-based learning [53]. The widespread use of AI has made chatbots popular for accessing health information.

This study found that information utility is one of the most valued attributes of AI chatbots among participants. Participants appeared to prefer chatbots that provide practical and relevant information, aligning with the findings of Kim [26] in their systematic review and meta-analysis on women’s health, which suggested that AI chatbots may positively impact women’s health by reducing anxiety and depression, promoting healthy behaviors, and offering health education. Although the review highlights that the provision of practical and relevant information appears to be a key factor in improving health outcomes for women, our current study found that the *P* value of the “less practical” level did not reach significance, indicating that the practicality of information only has a significant impact on user choices when it is very high or very low. This suggests that users expect the information provided by chatbots to have practical application value. Therefore, when designing chatbots, developers should consider focusing on providing practical information that can meet users’ actual needs and be immediately applied to their daily lives.

The results also indicated that response accuracy and readability significantly influence participants’ preferences. Participants seemed to favor AI chatbots with high accuracy and comprehensible content, which aligns with the systematic review by Aggarwal et al [54]. Aggarwal et al [54] included 15 empirical studies on AI chatbots that facilitate health behavior changes, including interventions like healthy lifestyles, smoking cessation, and medication adherence. While some studies demonstrated the efficiency of AI chatbots, mixed results were observed regarding their feasibility, acceptability, and usability [55,56]. These findings suggest that improving chatbot accuracy and readability is crucial for effectively promoting behavior change. Accurate health information is likely key to building user trust [57], and this study further confirms that participants valued accurate responses. Readability also plays a significant role in users’ choices, particularly among those with low health literacy or unfamiliarity with complex medical terms, as easily understandable information enhances comprehension and adherence [58-60]. In this study, the *P* values for the “difficult to understand” attribute were not statistically significant, suggesting that the readability of AI chatbot information might not substantially influence user preferences within certain thresholds. This may indicate that users prefer levels of readability that are “easier to understand” or “very easy to understand.” When information reaches a level deemed difficult to comprehend, users might be disinclined to engage further with the AI chatbot. These findings suggest that AI chatbots should aim to optimize language and interface design based on accurate content to ensure comprehensibility and cater to diverse women’s needs.

Regarding content preferences, participants showed a greater interest in disease knowledge, consistent with the systematic review by Younis et al [61], which categorized AI apps across health care domains such as patient support and education. AI chatbots appear to provide personalized health education and disease prevention information, offering content relevant to users’ needs. The preference for pregnancy and childbirth knowledge did not seem to be significant, indicating that these types of information do not have a clear advantage in user selection preferences. This may be because women are more concerned with the prevention, diagnosis, and treatment of diseases themselves, and may not necessarily have a direct need for information related to pregnancy and childbirth as much as disease knowledge. However, it is important to note that the high preference for disease knowledge could also be influenced by the fact that participants were recruited from a gynecology unit of a hospital, where individuals are likely to have one or more health conditions and a stronger interest in learning about diseases. We cannot conclusively assume that disease knowledge would rate as highly in a nonhospital sample, and this should be taken into consideration when interpreting the findings. While emphasizing the importance of providing information on disease knowledge, it is crucial not to overlook the demand for content in other areas. Women’s health is a multifaceted field that includes knowledge related to gender, pregnancy, childbirth, daily health care, and various diseases. Therefore, when developing chatbots, it is essential to consider all these aspects comprehensively to address the diverse needs of users.

On the topic of service cost, participants appeared to prefer free AI chatbots. This preference is consistent with other systematic reviews [62], which noted that free or low-cost services are more easily accepted by users, particularly when economic pressures are high. In a fast-paced era, the accessibility of free AI chatbots allows users to seek answers anytime and anywhere, increasing their reliance and usage. The preference for free services spans all age groups and socioeconomic backgrounds, suggesting that financial factors may strongly influence the choice for cost-free AI tools.

Finally, data collection did not significantly influence participants’ preferences. However, Kim [26] emphasized the importance of privacy protection in AI apps for women’s health. While data collection can help improve service quality, ensuring data security remains crucial in health care settings. Users generally value the protection of personal health data, and limiting data collection to nonidentifiable question and answer content can help alleviate concerns about data misuse or breaches.

This study provides valuable insights but has several limitations. First, it used convenience sampling and only included gynecological outpatients from a hospital in China. Therefore, the representativeness of the sample is limited. Since these female patients were surveyed in a medical setting, they are likely to be more interested in learning about diseases. As a result, the findings may not fully reflect the preferences and needs of a broader female population regarding women’s health chatbots. Therefore, future research should test these chatbot attributes in different cultural and social contexts to assess whether these preferences are universal or context-dependent. Second, the assumptions in the questionnaire might not fully reflect real-world preferences. Demographic factors such as age, education, and health literacy can influence how users engage with AI chatbots. Therefore, future studies should consider including a more diverse sample to capture a wider range of preferences. Finally, other variables, such as trust in technology and health care system perceptions, were not explored in this study but may impact user preferences. Expanding research to include these factors and diverse populations will help refine chatbot design to better meet the needs of different groups. Future studies should explore these aspects to provide a more comprehensive understanding of AI chatbot preferences across various contexts.

### Conclusions

This DCE highlights key preferences for AI chatbots in health care, indicating that users prioritize high response accuracy (100%), legibility, free services, and the practical utility of information, particularly about disease knowledge. These findings suggest that the development of AI chatbots should emphasize clarity, accessibility at no cost, user privacy protection, and the relevance of health content. By incorporating these attributes, user engagement can be enhanced, the dissemination of health education can be improved, and the effectiveness of AI tools in promoting disease management can be maximized.

## Abbreviations

AI: artificial intelligence
DCE: discrete choice experiment
WTP: willingness to pay
